# The Effects of Insufflation Conditions on Rat Mesothelium

**DOI:** 10.1155/2013/816283

**Published:** 2013-06-24

**Authors:** Andrew K. Davey, Jessica Hayward, Jean K. Marshall, Anthony E. Woods

**Affiliations:** ^1^Research Centre for the Molecular Basis of Disease, Griffith Health Institute, Griffith University, Gold Coast, QLD 4222, Australia; ^2^School of Pharmacy and Medical Science, University of South Australia, Adelaide, SA 5001, Australia; ^3^Fisher & Paykel Healthcare Limited, 15 Maurice Paykel Road, East Tamaki, Auckland 2013, New Zealand; ^4^Graduate School of Medicine and Illawarra Health and Medical Research Institute, University of Wollongong, Wollongong, NSW 2500, Australia

## Abstract

*Aim*. The aim of this investigation was to examine the alterations in the peritoneum after cold dry CO_2_, heated dry CO_2_, and humidified heated CO_2_ at pressures equivalent to intraperitoneal pressures used in human laparoscopy. *Methods*. Eighteen rats were divided into 4 treatment groups—group 1: untreated control; group 2: insufflation with cold dry CO_2_; group 3: insufflation with heated, dry CO_2_; group 4: insufflation with heated and humidified CO_2_. The abdomen was insufflated to 5 mm/Hg (flow rate 50 mL/min) for 2 h. Twelve hours later, tissue samples were collected for analysis by light microscopy (LM) and scanning electron microscopy (SEM). *Results*. Group 1: no abnormalities were detected. Group 2: specimens revealed an inflammatory response with loss of mesothelium and mesothelial cell nuclei showing lytic change. Cells were rounded with some areas of cell flattening and separation. Group 3: some animals showed little or no alteration, while others had a mild inflammatory response. Mesothelial cells were rounded and showed crenation on the exposed surface. Group 4: specimens showed little change from the control group. *Conclusions*. The LM results indicate that insufflations with heated, humidified CO_2_ are the least likely to induce mesothelial damage.

## 1. Introduction

Carbon dioxide (CO_2_) gas is the most common insufflation agent used to create pneumoperitoneum during laparoscopic surgery [1]. In the absence of conditioning, standard CO_2_ used in laparoscopic surgery is cold and dry at 20 to 21°C, <1% relative humidity, at the point of entry into the peritoneal cavity [2, 3]. The condition of standard CO_2_ is in marked contrast to the physiologic intra-abdominal condition. Experimental and clinical investigations have demonstrated that the cool, dry nature of standard CO_2_ insufflation causes desiccation resulting in visible structural, morphological, and biochemical alterations to the mesothelial cells of the peritoneum [4–8]. This includes bulging of the mesothelial cells, widening of intercellular junctions, exposure of the basement membrane [5], and increased peritoneal cytokine response [9]. Previous animal investigations have demonstrated that the use of humidified heated CO_2_ can attenuate peritoneal damage caused by desiccation [4, 6]. Compared to standard CO_2_ insufflation, the use of humidified heated CO_2_ results in no exposure of the basement membrane [4], no visible intercellular clefts [4], improvements in perioperative temperature, and decreased adhesion formation [6]. However, insufflation during these animal investigations was performed in rodent models at pressures greater than 8 mmHg. The more recent literature indicates that pneumoperitoneal pressures ≥8 mmHg in a rat model correlate to high intraperitoneal pressures in humans, greater than the standard working pressures [10]. An excessively high intraperitoneal pressure may cause detachment of mesothelial cells by a mechanical effect, severe hypoxia, or both [11]. In addition, these studies fail to assess the effect of heated-only CO_2_ on the peritoneum. The aim of this investigation was to examine the alterations of the peritoneum after standard cold dry CO_2_, heated dry CO_2_, and humidified heated CO_2_ at pressures equivalent to intraperitoneal pressures used in human laparoscopy.

## 2. Materials and Methods

### 2.1. Animal Protocol

This study was approved by the Institute of Medical and Veterinary Science (IMVS) Animal Ethics Committee (Adelaide, SA, Australia). Male Sprague-Dawley rats were obtained from the Animal Resource Centre (Gilles Plains, SA, Australia) and kept in individual cages on a 12 h light-dark cycle, with free access to standard diet and water in a room at a temperature of 20 to 23°C. They were divided into treatment groups:

 group 1—control: no insufflation, anaesthesia only (*n* = 3);
 group 2: insufflation with cold (room temperature), dry CO_2_ at a pressure of 5 mmHg (*n* = 5);
 group 3: insufflation with heated (37°C), dry CO_2_ at a pressure of 5 mmHg (*n* = 5);
 group 4: insufflation with heated (37°C) and humidified (100% RH) CO_2_ at a pressure of 5 mmHg (*n* = 5).

CO_2_ in groups 3 and 4 was conditioned using the Fisher & Paykel Healthcare MR860 Laparoscopic Humidification System (LHS) (Fisher & Paykel Healthcare, Auckland, New Zealand). Under standard use, as per study group 4, the insufflation gas passes over a heated humidification chamber filled with 30 mL of sterile water which is designed to condition the gas to 37°C and 100% RH [12]. The condition of the gas is maintained as it flows down the humidified insufflation tube which acts to maintain the condition of the gas direct to the laparoscopic port. The system monitors the temperature and flow rate of the gas and automatically alters the heater-plate temperature to maintain consistent gas conditions. For group 3, the system was used off-label as no water was added to the humidification chamber resulting in warm (37°C), dry insufflation gas.

On the day of the experiment, the rats were anaesthetised with inhaled isoflurane. Prior to use, gas was passed through the LHS until reaching 37°C. The gas flow rate was continually measured using a thermal mass flow meter (“red-y smart series”, Vogtlin Instruments AG, Aesch, Germany) calibrated for use with CO_2_ gas. The flow rate was adjusted to 50 mL/min via a flow restrictor (Precision Flow Control Valve, GRPO-10-PK-3, Esslingen, Germany). The target flow rate of 50 mL/min was calculated according to the average peritoneal surface area of the experimental rats [13]. The abdomen was insufflated (CO_2_-OP-Pneu Insufflator, Wisap, Munich, Germany) to 5 mm/Hg through a 16 G port site cannula inserted into the side of the lower abdomen. When there was no gas flow, a 26 G exit port cannula was inserted into the opposite side of the abdomen. Rats were kept under anaesthesia for 2 hours. Body temperature was measured with a rectal thermometer during insufflation and normothermia maintained using a warming pad beneath the animal. The temperature was recorded every 15 min, and no significant changes in body temperature were observed. At the end of the 2 h experimental period, the CO_2_ was turned off and disconnected. The rats were allowed to rest for 1 to 2 min to allow gas to escape through the cannulae. Once the abdomen had finished deflating, the cannulae were gently removed and the abdominal wall closed with surgical silk. Following surgery, the anaesthetic was switched off, and the rats were allowed to recover. 

### 2.2. Tissue Collection and Analysis

Twelve hours after the completion of surgery, the rats were again anaesthetised with isoflurane. After opening the abdomen, tissue samples were collected at several sites along the abdominal wall. Only samples collected away from the insertion sites were used in order to avoid physical trauma from the incision or the cannula confounding the results. Rats were euthanised immediately after tissue sample collection. Specimens were collected from each study group and fixed by immersion for at least 24 h in 10% buffered formalin for examination by light microscopy (LM) or 2.5% glutaraldehyde for scanning electron microscopy (SEM).

### 2.3. Light Microscopy

Fixed samples were processed in an automated processor (LEICA ASP300S, Leica Microsystems, Wetzlar, Germany) into paraffin wax using a routine schedule (70% ethanol, 90% ethanol, absolute ethanol, xylene, and paraffin wax). 

Paraffin sections were cut at 4 *μ*m thickness using a Microm (HM 325, Walldorf, Germany) microtome and collected onto slides. Sections were stained using a standard H&E procedure, coded, and examined blind to the code used. Inflammatory cells (neutrophils, eosinophils, and macrophages) were identified morphologically. The inflammatory cell concentration along the mesothelium and submesothelial lamina propria was determined according to the following semiquantitative scale: +, 0–5 cells per high power (×40 objective) field (hpf); ++, 6–15 cells per hpf; +++, >15 cells per hpf. Micrographs were captured using an Olympus BX40 microscope (Olympus, Hamburg, Germany) fitted with a DP70 digital camera linked to Olympus Cell^∧^B Imaging Software (Olympus Soft Imaging Solution GmbH, Munster, Germany).

### 2.4. Scanning Electron Microscopy

Fixed samples were washed in 0.1 mol/L cacodylate buffer then postfixed in 1% osmium tetroxide for 90 min. Following a further wash in the buffer, samples were dehydrated through a graded alcohol series to super dry alcohol then completely dried in a critical-point drying apparatus using liquid CO_2_ as the exchange medium. Dehydrated specimens were mounted onto aluminium stubs then coated with carbon/gold in a Denton DV-502 Vacuum Coater (Denton Vacuum, LLC, New Jersey, USA). Tissue specimens were examined using a Philips XL20 SEM (Philips, Eindhoven, The Netherlands) operated at 10 kV. The spot size was recorded directly on the images.

## 3. Results

### 3.1. Light Microscopy

**Figure 1:**
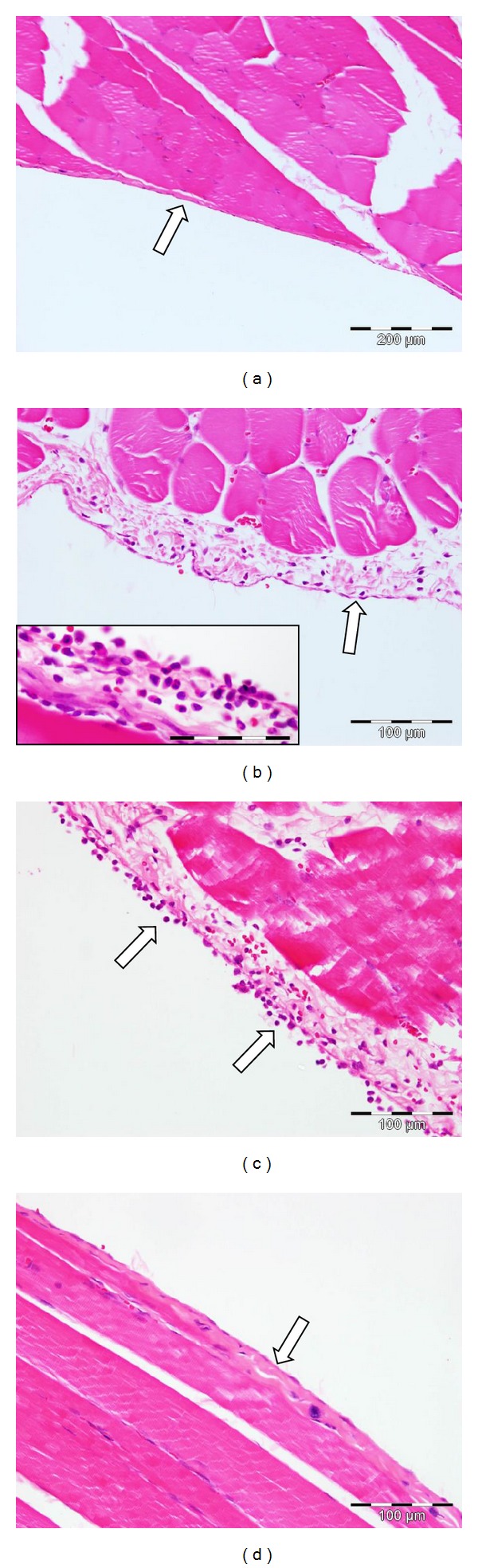
Selected light micrographs showing the mesothelial surface 12 h after insufflation with CO_2_ for 2 h at 5 mm/Hg of (a) control animals, no treatment with CO_2_; (b) insufflation with room-temperature, dry CO_2_ where inflammatory cells are evident in the lamina propria. Inset: inflammatory cells on surface of mesothelium (scale bar = 100 *μ*m); (c) insufflation with heated, dry CO_2_. Inflammatory cells are evident on the mesothelial surface; (d) insufflation with warm, humidified CO_2_ with normal mesothelium. Arrows indicate the mesothelial surface.

Light microscopy observations are detailed in Table 1. A number of sections showed some level of physical damage, drying and/or stretching artefact due to the sample collection process (data not shown). Within group 1 (control) no tissue changes were detected in any specimen (Figure 1(a)). 

Following insufflation with cold (room temperature), dry CO_2_ (group 2), most specimens revealed an inflammatory response indicated by a mixed population of acute inflammatory cells consisting of neutrophils, eosinophils, and macrophages (Figure 1(b)). The extent of the inflammatory infiltrate was variable ranging from very few cells to an intense inflammatory response which extended to both sides of the mesothelial surface (Figure 1(b) inset). Loss of the mesothelium was evident in some areas.

Variable results were noted in group 3 (heated and dry). Some animals showed little or no alteration to the mesothelium, while in others a mild nonspecific inflammatory response was observed. Rat C (Figure 1(c)) displayed isolated areas which exhibited a more prominent inflammatory infiltrate and rounded mesothelial cells. The changes observed might reflect the changes to the intra-abdominal environment but this cannot be confirmed without additional studies. 

The morphology of the samples exposed to heated and humidified CO_2_ (group 4) was similar to that in the control group: the mesothelium was intact, and there was little inflammatory cell infiltrate (Figure 1(d)). The latter noted in rats B and C may reflect the changes to the intra-abdominal environment but this cannot be confirmed without additional studies.

### 3.2. Scanning Electron Microscopy

The changes identified by SEM mirrored the changes noted by LM although inflammatory cells were not seen in any of the specimens examined. As these cells are not usually firmly anchored in tissues, the likelihood is that any cells that were present were unintentionally removed from the surface during preparation.

**Figure 2:**
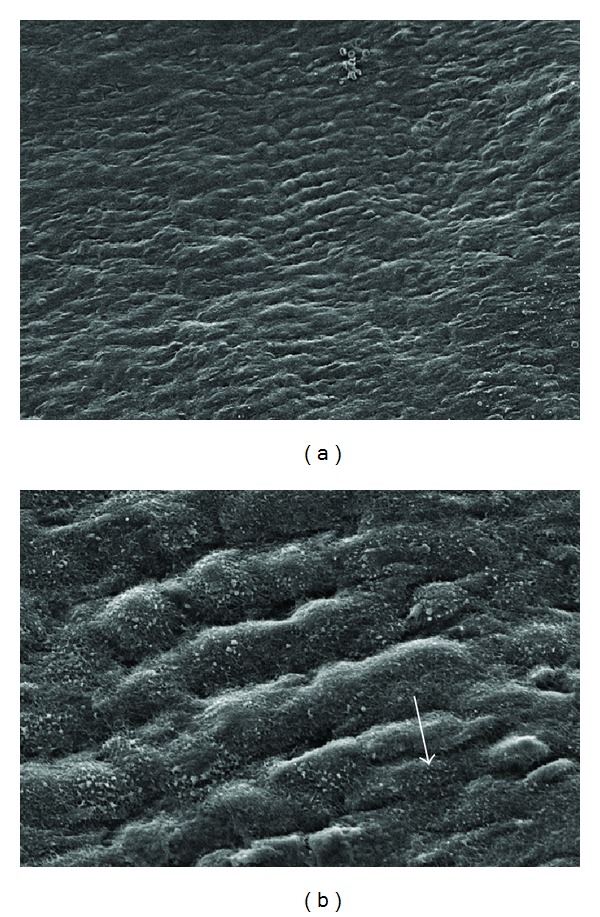
SEM showing mesothelial surface from control animals (group 1). Cells are aligned in rows with some elevation; microvilli are present but sparse (arrow): (a) original ×650; (b) original ×3500.

The control samples showed cells aligned in rows with some elevation (Figure 2). Microvilli were scantly present which is a characteristic of the flat mesothelial cells of the parietal peritoneum [14]. 

**Figure 3:**
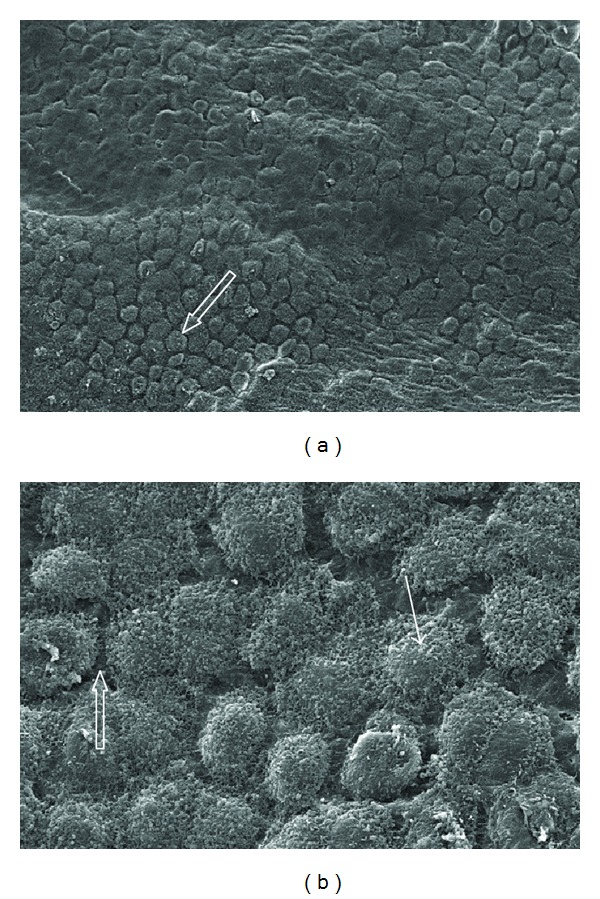
SEM showing mesothelial surface from rats 12 h after insufflation with room-temperature, dry CO_2_ for 2 h at 5 mm/Hg (group 2). Cells are distinctly rounded with some areas of cell flattening and separation (open arrow). Microvilli are collapsed and indistinct (arrow). Some invagination of the tissue suggestive of dehydration is evident in some regions: (a) original ×650; (b) original ×3500.

Group 2 (room-temperature, dry CO_2_) cells were distinctly rounded with some areas of cell flattening and separation (Figure 3). Microvilli were collapsed and poorly defined. Some invagination of the tissue suggestive of dehydration was evident in occasional regions.

**Figure 4:**
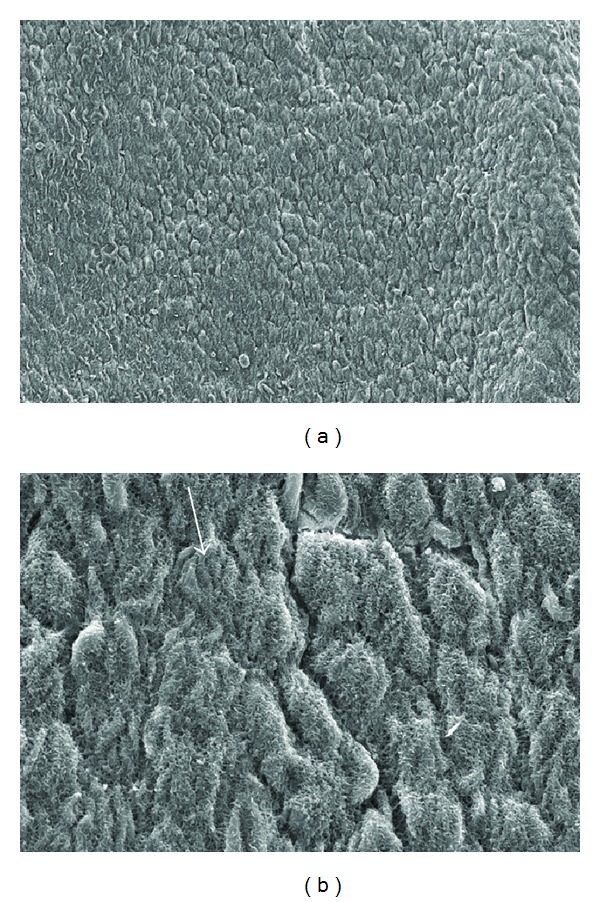
Mesothelial surface from rats 12 h after insufflation with warm, dry CO_2_ for 2 h at 5 mm/Hg (group 3). Mesothelial cells are rounded and show distinct indentations (crenation) to the exposed surface (arrow): (a) original ×650; (b) original ×3500.

**Figure 5:**
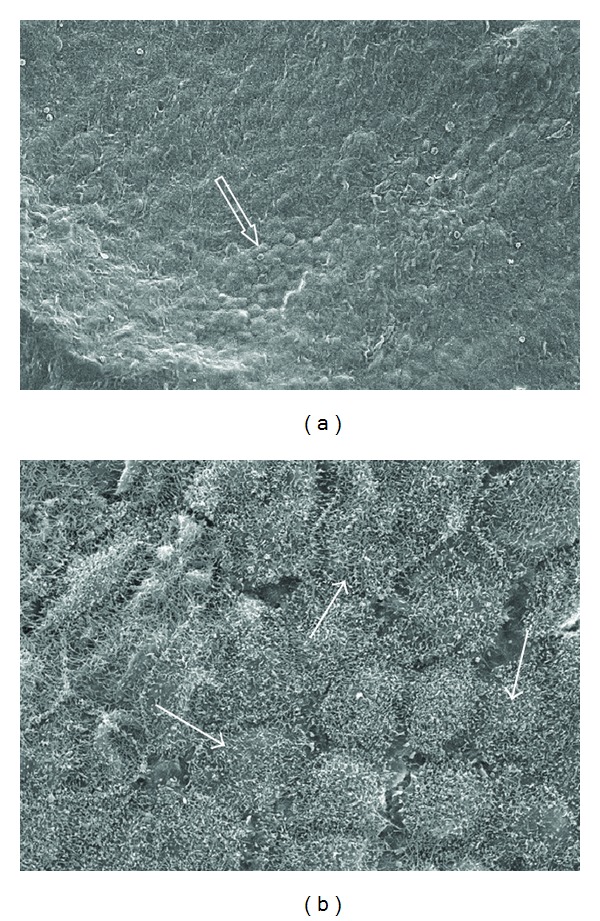
Mesothelial surface from rats 12 h after insufflation with heated humidified CO_2_ for 2 h at 5 mm/Hg (group 4). The cells are tightly apposed with cytoplasmic projections (microvilli) prominent (arrows). While some rounding of the cells is evident (open arrow), overall this group showed the least effects from the induced environment: (a) original ×650; (b) original ×3500.

In group 3 (warm, dry CO_2_), mesothelial cells were rounded and showed distinct indentations (crenation) on the exposed surface (Figure 4). This was probably due to exposure to the dry environment. Within group 4 (humidified, heated CO_2_), the cells were closely apposed with cytoplasmic projections (microvilli) prominent (Figure 5). While some rounding of the cells was evident, overall this group showed similar morphology to the control samples indicating it exhibited the least effects from the induced environment.

## 4. Discussion

The use of insufflating gas facilitates laparoscopy by lifting the abdominal wall and creating a working space [1]. The ideal gas would be nontoxic, odourless, colourless, highly soluble, readily excreted by the lungs, and noncombustible/does not support combustion and inexpensive. Insufflation gases currently in use all have limitations. Helium and argon are inert but poorly soluble which leads to concerns relating to potential embolism and pneumothorax; nitrous oxide can explode when using electrocautery; and CO_2_ may cause local and systemic acidosis and cardiorespiratory effects (reviewed by Neuhaus et al., 2001 [1]). Of these gases, CO_2_ is by far the most commonly used, its major advantages over the alternatives being that it is very soluble and rapidly excreted. 

Despite the advantages of using CO_2_ as an insufflation gas in laparoscopic surgery, it has been associated with pain, including during awake laparoscopy [15], postsurgery shoulder-tip pain [16], adhesion formation [6, 17], and increased susceptibility to cancer metastasis [18]. Most of these adverse effects are linked to damage and/or inflammatory activity to the peritoneal mesothelium, and there are several putative mechanisms by which pneumoperitoneum can cause mesothelial damage. CO_2_ dissolves in water to form carbonic acid which is suggested to lead to peritoneal acidosis [19]. Alternatively, if the gas is used at high pressure, it can increase airway pressure [10] and reduce cardiac output and peritoneal blood flow leading to acidosis, hypoxia, and oxidative stress [11, 20]. This is a particular concern when extrapolating experimental results from small animal models, where disproportionally high insufflation pressures are often used [10, 11]. The condition of the gas is also a factor. Generally, CO_2_ is administered at room temperature (cold) and at very low relative humidity (dry). Cold gas is associated with hypothermia which is linked to numerous postoperative problems [21], although some slight cooling in the absence of desiccation may be protective [17], presumably due to suppression of the immune system and reduction of the metabolic activity of the cells. Using dry gas can damage the mesothelium also due to water evaporating from the peritoneal surface causing desiccation of the cells and further contributing to the hypothermia through the evaporative cooling effect [17, 21, 22]. The insult to the mesothelium through a combination of these mechanisms induces an inflammatory response which further acts to damage the tissue including sloughing of cells [7], which increases susceptibility to cancer spread [23] and adhesion formation [24]. 

The extent of mesothelial damage would be expected to increase with longer duration of pneumoperitoneum as well as with higher pressures and flow rates [22, 25, 26]. Hence, any protective effect through conditioning of the gas should become more apparent under those conditions where damage is likely to be substantial (i.e., long duration, high pressure, and high flow rate). The use of heated gas may have some protective effects against hypothermia and inflammation [9]. However, the dominant mechanism by which gas insufflation causes heat loss is through evaporative cooling [27]. When the gas is heated without humidification, it has an even greater potential to cause evaporation than unheated gas that can lead to increased tissue cooling and to much greater tissue desiccation. This may explain why heated gas may be associated with increased postoperative pain [28]. Humidification of the CO_2_ as well as heating can deliver gas to the peritoneum that should not cause desiccation. Care does need to be taken to ensure that the temperature/humidification combination is correct such that condensation of water does not occur in the peritoneum, which causes osmotic shifts [27]. Heating and humidification can reduce pain and improve postoperative recovery [29–31] by a number of potential mechanisms. Glew et al. demonstrated that residual gas dissipated more quickly after surgery in piglets when it was humidified [32]. This was attributed to the preservation of the moist milieu of the serous fluid lining of the peritoneal cavity, enabling the CO_2_ to rapidly dissolve. Within that study, humidification was also associated with a reduced systemic cytokine response. The ability of the CO_2_ to dissolve and dissipate rapidly may reduce the potential for localised acidosis too. Despite their ultimate conclusion, Wong et al. demonstrated a more acidic peritoneum when using standard cold dry gas compared to heated and humidified (*P* < 0.05) in pigs [19]. Reduction of evaporation by humidifying the gas significantly reduces the potential for hypothermia as well [6, 21]. However, the greatest benefit of heated and humidified gas potentially rests in the prevention of mesothelial damage through desiccation and the associated inflammatory response. But there is a paucity of studies that investigate this in animal models, and of those the majority uses insufflation conditions likely to cause exaggerated damage due to the high pressures, flow rates, and long durations of pneumoperitoneum [4, 6, 10, 33]. Furthermore, direct comparisons between heated/humidified, heated/dry, and cold/dry CO_2_ are needed to elucidate the effects of the different combinations on the peritoneum.

Within the current study, our aim was to use conservative conditions to compare the effects of the three different temperature/humidity combinations on mesothelial cells. The 2 h pneumoperitoneum was chosen as a moderate time with the pressure within the criteria recommended by Avital et al. [10]. In addition, the use of a heating blanket to maintain normothermia reduced the potential impact of hypothermia as a confounding variable in our study. Previously it has been demonstrated that mesothelial changes of the type we were investigating are most apparent at 12 h after surgery [7], and so this was used to compare whether heating and/or humidification had any protective effects. 

Our results strongly suggest that under the conditions of the study, cold/dry CO_2_ caused mesothelial damage comparable to that previously reported [7], with little protection provided by heating the gas. In contrast, heating with humidification protected the mesothelial cells such that there was little deviation from the control animals. This is supported by the findings of other studies albeit under more extreme experimental conditions [4, 6]. In contrast, Hazebroek et al. found no difference between warm/dry, cold/dry, and warm/humidified CO_2_ [34]. There are a number of potential reasons for this. Hazebroek et al. used a 2 h and a 24 h time point to sacrifice the animals and obtain the tissues rather than the 12 h time point used in our study. In addition, the different methods of anaesthesia may have impacted the findings. We used inhaled isoflurane to avoid any direct irritation within the peritoneum from the anaesthetic whereas Hazebroek et al. used intraperitoneal pentobarbitone administered every 30 min. Pentobarbitone is known to have irritant effects [35] which may have significantly contributed to the mesothelial damage. This hypothesis is supported by the fact that the group with mechanical abdominal wall lifting in the study showed similar damage to the three insufflation groups, indicating that pneumoperitoneum was not the cause of the mesothelial damage.

The results from this study demonstrate that heated and humidified CO_2_ provides much greater protection of the mesothelium compared to either heated or cold dry gas in rats at insufflation pressures and times comparable with human surgery.

## Figures and Tables

**Table 1:** Summary of light microscopy (LM) observations 12 h after insufflation with CO_2_ for 2 h at 5 mm/Hg.

Group 1	
A	NAD
B	NAD
C	NAD
Group 2	
A	NAD
B	Inflammatory cell infiltrate (++) Fragmentation and desquamation of mesothelium in some sections
C	Inflammatory cell infiltrate (+++) Some inflammatory cells migrated onto mesothelium surface
D	Inflammatory cell infiltrate (+)
E	Inflammatory cell infiltrate variable in concentration along mesothelium (+ to +++) Inflammatory cells migrated onto mesothelium surface
Group 3	
A	Inflammatory cell infiltrate (+++) with cells on mesothelial surface Loss of mesothelium in some sections of intense inflammatory cell infiltration Remaining areas uninvolved
B	Scant areas of inflammatory cell infiltrate (+)
C	Inflammatory cell infiltrate (++) Mesothelial cell nuclei rounded and projecting above mesothelial surface
D	Scant areas of inflammatory cell infiltrate (+)
E	Scant areas of inflammatory cell infiltrate (+)
Group 4	
A	Patches of inflammatory cell infiltrate (+)
B	Inflammatory cell infiltrate (+ to ++)
C	Patches of inflammatory cell infiltrate (+ to +++)
D	Patches of inflammatory cell infiltrate (+)
E	NAD

Group 1: control animals, no treatment with CO_2_. Group 2: insufflation with room temperature, dry CO_2_. Group 3: insufflation with warm, dry CO_2_. Group 4: insufflation with warm, humidified CO_2_. Letters (A–E) refer to individual rats. NAD: no abnormality detected.
